# Comparing community-based interventions versus population-wide response in information diffusion on social media platforms

**DOI:** 10.1017/dap.2025.10053

**Published:** 2026-01-22

**Authors:** Chathura Jayalath, Xiaoxia Champon, William Rand, Jasser Jasser, Ozlem Garibay, Ivan Garibay

**Affiliations:** 1 Industrial Engineering and Management Systems, University of Central Florida, Orlando, FL, USA; 2 Statistics, NC State University, Raleigh, NC, USA; 3 Poole College, NC State University, Raleigh, NC, USA; 4 Mathematics and Computer Science, Rollins College, Winter Park, FL, USA

**Keywords:** functional data analysis, group differences, community-based interventions, social media

## Abstract

The dynamics of information diffusion on social media platforms vary significantly between individual communities and the broader population. This study explores and compares the differences between community-based interventions and population-wide approaches in adjusting the spread of information. We first examine the temporal dynamics of social media groups, assessing their behavior through metrics such as time-dependent posts and retweets. Using functional data analysis, we investigate Twitter activities related to incidents such as the Skripal/Novichok case. We present three ways to quantify disparities between communities and uncover the strategies used by each group to promote specific narratives. We then compare the impact of targeted, community-based interventions with that of broader, population-wide responses in shaping the diffusion of information. Through this analysis, we identify key differences in how communities engage with and amplify information, revealing distinct patterns in the diffusion process. Our findings provide a comparative framework for understanding the relative consequences of different intervention strategies, offering insights into how targeted and broad approaches influence public discourse across social media platforms.

## 1. Introduction

Social media has become increasingly prevalent as a communication tool and a valuable source of fresh conflict-related data (Zeitzoff, 2017). The rapid dissemination of information and the sheer volume of exchanges necessitate unique strategic responses, ideally timed to address emerging issues. To this end, certain organizations, often funded by public resources, are established to define and oversee what serves the public’s best interests (Bradshaw and Howard, 2017). Moreover, the influence of manipulation and fake news within private groups can be just as potent as in traditional communication reliant on opinion leaders (Zakharchenko et al., 2019). The importance of a suitable response from public officials on social media cannot be overstated. Timely engagement with carefully crafted messages can effectively harness resources to enhance services and foster improved communication with citizens (Kavanaugh et al., 2011).

Badawy et al. (2020) investigated the significant impact of manipulation campaigns on event outcomes, revealing that users who frequently shared posts from Russian troll accounts on Twitter often belonged to specific communities. Similarly, Gunaratne et al. (2020) affirmed the constraining influence of information overload on the online behavior of social media users, thereby affecting the population-level dynamics of information dissemination during online discussions. It becomes crucial to discern posting disparities and the ripple effect of retweets within these community groups, as they ultimately determine the outcomes of orchestrated campaigns. Such insights can prove invaluable in shaping time-sensitive response strategies, particularly when dissemination occurs through a strategy like “Flooding-The-Zone” (FTZ) as defined by Jasser (2023). Our objective is to systematically comprehend the behavioral distinctions among diverse social media communities and pinpoint key characteristics that can facilitate more efficient and meaningful differentiation between these groups. These distinctions can then inform more appropriate and effective engagement strategies as outlined in the group intervention and population response section.

Our research draws inspiration from a report published by the Policy Institute at Kings College London (Ramsay and Robertshaw, 2019) that investigated the weaponization of RT and Sputnik news channels, along with social media, in disseminating disinformation regarding the poisoning of former Russian double agent Sergei Skripal and related campaigns. In a similar vein, Wagnsson (2023) conducted an examination of Russian messaging, utilizing RT/Sputnik as channels for the dissemination of malign information. For our study, we inspect Twitter data pertaining to the Skripal case (Poisoning of Sergei and Yulia Skripal, 2023) spanning from March 4 to April 30, 2018, with the aim of exploring the pathways of influence within social media communities.

The data observed for each Twitter user including their user details, tweets containing keywords such as “Skripal” and “Novichok” (referring to the nerve agent used) for the Skripal case. Additionally, the dataset includes links to 1000 relevant online news articles. Our research focuses on exploring specific differences among social media groups by analyzing the number of posts and retweets made by users over a 60-day period for the Skripal case. Our work follows (Champon et al., 2024) and draws from functional data analysis (FDA) to examine the temporal patterns of user postings and retweets related to major incidents such as the Skripal/Novichok case. By comparing the behavior of various social media groups, we seek to identify critical differences in how information flows within these communities and across the broader population. This research aims to offers valuable perspectives for policymakers on the differences between community-based interventions versus population-wide approaches, particularly in managing the spread of disinformation.

Champon et al. (2024) conducted a constructive analysis on the disparities between these two opposing groups that shed light on invaluable in discerning the pathways through which information flows. It quantified the disparities between these communities and uncovered the strategies employed by each group to promote specific campaigns. Therefore, community-based interventions and population-wide approaches are two natural strategies for interventions, particularly for public health. For example, while targeted interventions focus on high-risk individuals, population-wide approaches aim to impact the entire community (Ahern et al., 2008). Community intervention trials have shown potential benefits, including generalizability and efficiency in reaching large populations (Fortmann et al., 1995). However, design limitations and unexpected changes have made it challenging to draw definitive conclusions about overall effectiveness (Fortmann et al., 1995). Community-based interventions can target multiple levels of influence, address social inequalities, and involve communities in planning and implementation (Sorensen et al., 1998). In addition, agent-based modeling can be used to study information diffusion and optimize intervention delivery under varying conditions (Lindau et al., 2021). (Lindau et al., 2021) found that the spread of community resource information via social sharing was nearly four times greater than that of clinical delivery alone, highlighting the potential for interventions to have amplified effects through community diffusion. We intend to explain the differences between community interventions and population-wide approaches in the context of information diffusion under the FTZ framework.

The rest of this article is organized as follows. We briefly explain the methodology, which involves three techniques to differentiate social media communities, along with the corresponding results. We then present our findings and include a discussion section on different response strategies.

## 2. Methods

Our analysis comprises three key steps: to begin, we assign users to communities using the engagement network approach outlined by Jasser (2023) in their work. Subsequently, we employ Functional Principal Component Analysis (FPCA), as proposed by Ramsay and Silverman (2005), to compare the time-dependent patterns of posts and retweets between the two groups. Finally, we utilize the multivariate functional data clustering method, as detailed by Ren et al. (2023), which incorporates adaptive density peak detection techniques, to cluster users. We also investigate the canonical correlations between posts and retweets as part of our analysis. The subsequent subsections provide detailed coverage of each step.

### 2.1. Girvan–Newman algorithm to label communities

We started with three groups of total 3,576 users where 2,332 are pro-Russian, 1,138 are pro-Western, and 106 are neutral from Jasser (2023). These labels are calculated with Girvan–Newman algorithm with the girvan_newman function in the python library Networkx (Girvan and Newman, 2002; Hagberg and Conway, 2020) using the engagement network defined by the interactions between users on Twitter (Jasser, 2023).

In our study, we define Twitter users’ engagement as encompassing “retweeting,” “replying,” or “quoting” interactions between two users. We establish a network of users who have engaged with each other, where individual users are represented as nodes, and the interactions between them are depicted as edges. Whenever a user engages with any post from another user, we add an edge to connect these two users in the network.

**Figure 1.**
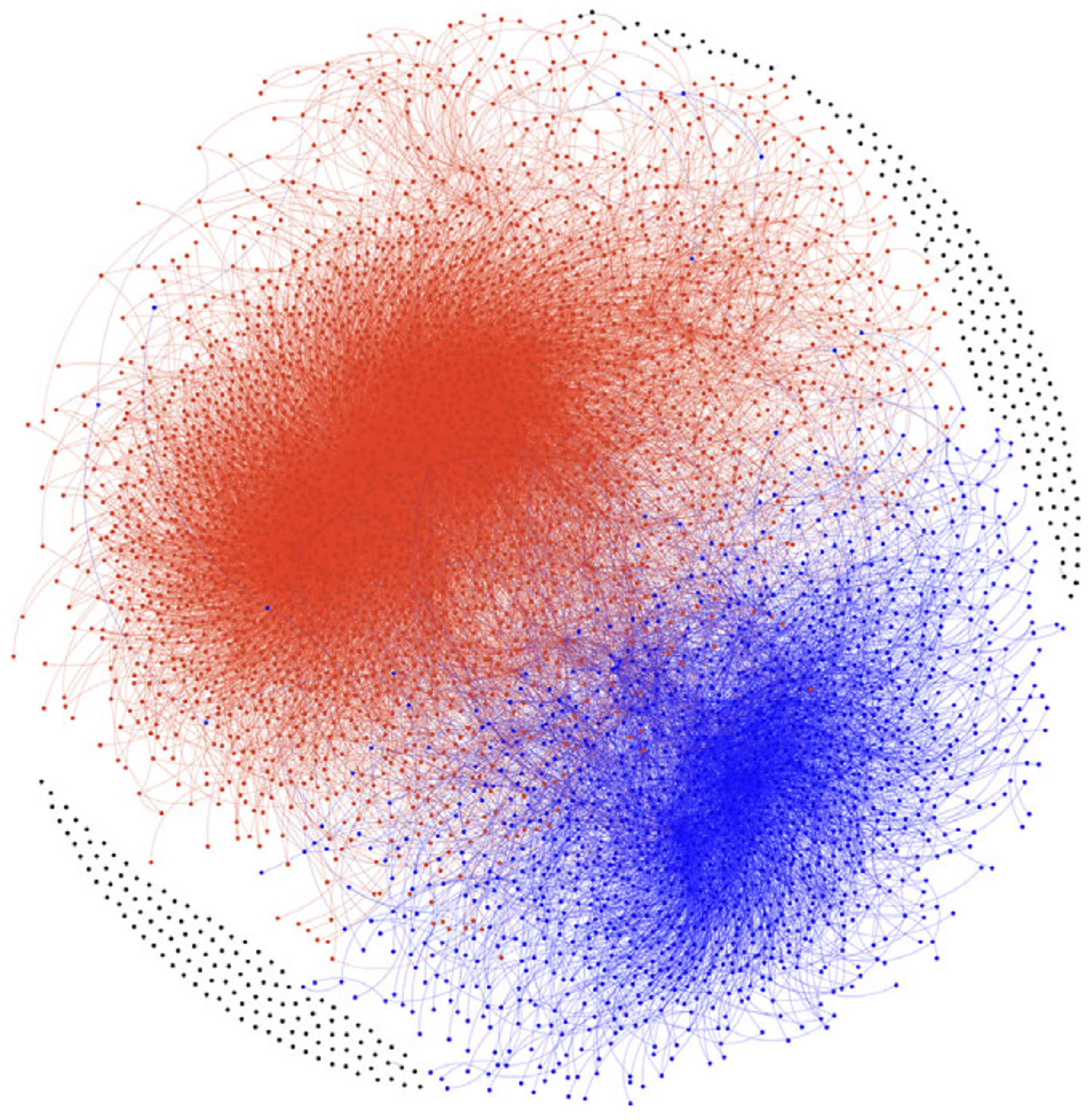
Users’ Engagements Network. The red echo chamber represents the Russian media side of the narrative (All the users who propagate the Russian narrative) while the blue echo chamber represents the Western media side of the narrative (Jasser, 2023).

These engagements among users on Twitter result in two prominent communities. One community is characterized by echo chambers created by the FTZ campaign, with the definition of “pro-Russian.” The other community opposes this narrative and is defined as “pro-Western,” as illustrated in Figure 1, where only nodes with a degree of 5 or more are displayed.

This community classification is particularly relevant because both incidents under scrutiny are closely associated with Russia and were top news stories at the time. We anticipate that top users from each community engage in posting messages on social media to advance agenda-based campaigns. Modeling the posting and retweet behaviors of these users can offer valuable insights into the characteristics of these communities. Note that while we identify these communities based on engagement between users, the motives of the users may remain distinct (An, Quercia, and Crowcroft, 2014). Our analysis focuses on exposure and propagation within polarized interaction structures, rather than assuming belief homogeneity.

### 2.2. Functional data analysis

In our analysis, functional data are characterized by the quantity of posts or retweets made by users over a closed time interval. The curves representing a user’s posts or retweets typically exhibit variations in both amplitude and phase across different observations. The primary objective of FDA is to delve into the behavior of these subject-specific curves. This includes quantifying the variability between the curves with respect to the mean curves, and examining the dependencies among observations within a subject.

Our approach to assessing the distinctions between social media communities hinges on exploratory investigations, primarily facilitated by techniques such as FPCA and Functional Canonical Correlation Analysis (FCCA; J. O. Ramsay and Silverman, 2005). We follow (Champon et al., 2024) to apply functional data methodologies to study information transmission on social media, especially in terms of tweets and retweets curves immediately following significant incidents. We then use simulation to reflect the impact of different response approaches.

#### 2.2.1. Data processing

We selected the same users from (Champon et al., 2024) for the FDA. A significant reduction of nearly 77% in the user pool from the original data was observed during this process. This reduction highlights the fact that while the engagement network may be extensive, it comprises a limited number of meaningful nodes or individuals who actively contribute significantly to the campaign agenda (Senevirathna et al., 2021).

The removal of noisy subjects or nodes is strongly recommended for any subsequent analysis, as it enables the detection of genuine signals in a more efficient manner (Senevirathna et al., 2021). This practice proves especially beneficial when information on different events over time circulates within similar communities involving the same individuals.

We used a total of 183 pro-Russian users and 364 pro-Western users in the tweets dataset (Jayalath et al., 2025). The same filtering criteria were applied to the retweets data, yielding 113 pro-Russian users and 159 pro-Western users for the Skripal incident.

This current ratio of individuals from each group markedly differs from our initial dataset. This significant shift can largely be attributed to the dynamics of censorship on Twitter and other social media platforms during that specific time period.

It is important to note that the users selected for the subsequent analysis played a substantial role in shaping the information flow on social media platforms. As such, our focus in the following analysis centers on these highly influential representatives.

As indicated in Table 1, it is evident that the pro-Western group has more than twice as many members as the pro-Russian group for these specific campaigns after applying the filtering conditions. It is worth noting that 38% of the tweets from pro-Russian users regarding Skripal are retweeted, with a significant 62% of pro-Russian group members contributing to a substantial volume of tweets. In general, the pro-Russian side tends to post more, attributed to the FTZ disinformation campaign, as discussed in Jasser (2023). This increased posting is a characteristic of FTZ campaigns that involve targeted disinformation efforts.

In contrast, tweets from the pro-Western group are retweeted only 30% of the time during our 60-day observation period for Skripal data. To be specific, terms like “Skripal” tend to attract more members from the pro-Russian camp than from the pro-Western camp. However, the focus of our work on identifying group differences centers on active users involved in multiple events. While this approach potentially limits the user sample to those with a high degree of involvement in both incidents, it allows us to identify users who consistently engage and form a potentially sample of highly engaged users for each community. This characteristic is likely to hold true for other time-varying campaigns as well.

Furthermore, we assume that the fundamental patterns of posting and retweets remain similar across different events, particularly in terms of the time it takes for response tweets to occur and for retweets to be exchanged, as these aspects are assumed to be closely related.

**Figure 2.**
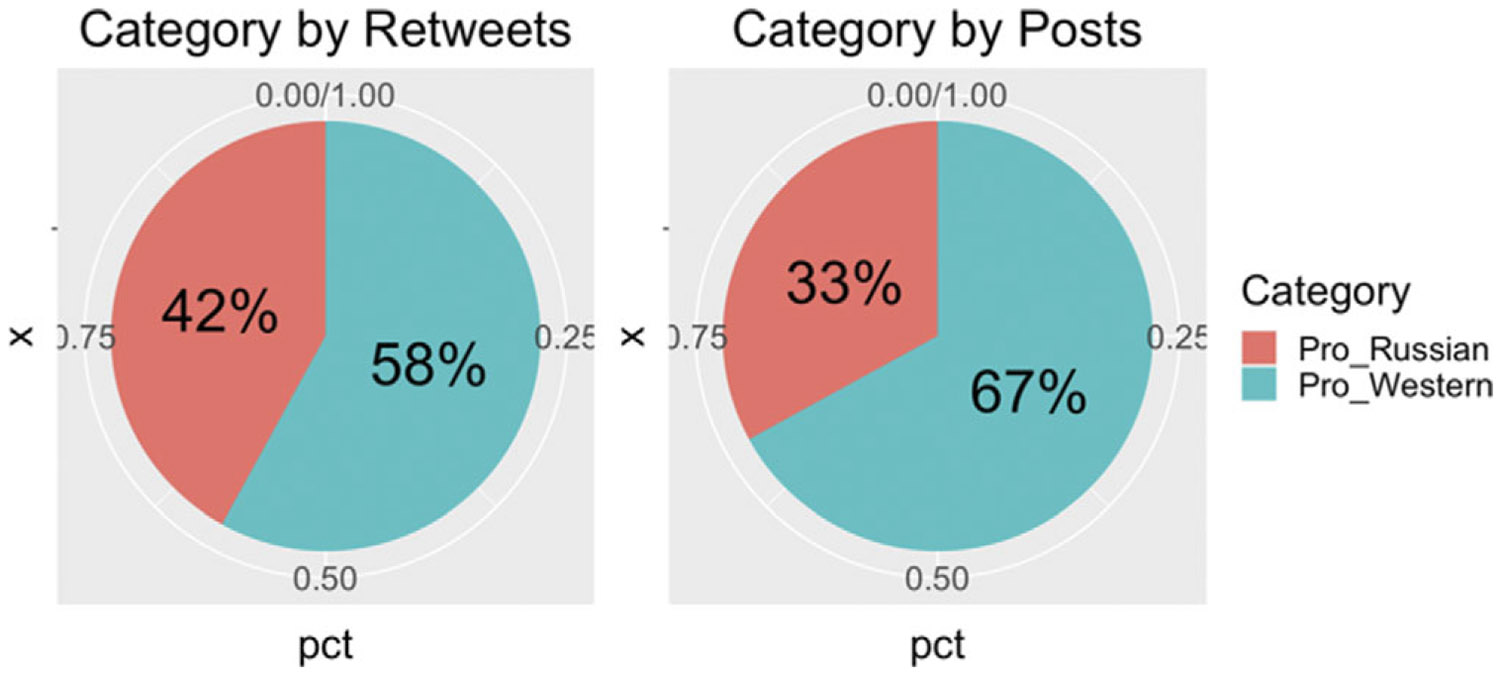
Skripal users distribution by posts and retweets.

The distribution of posts and retweets for the Skripal incident is shown in Figure 2. On average, the number of pro-Russian users increases engagements by 9% from posts to retweets for the Skripal incident. In general, the pro-Russian group demonstrates a longer-lasting commitment to the “Skripal” campaign, as anticipated in this study. Their activity intensifies not only in terms of tweet volume but also in the volume of retweets, despite the fact that users from this group make up less than half of the total population. In contrast, pro-Western group tweet more and less users tend to retweet the message for Skripal incident. Additionally, in the pro-Russian group, there are fewer users who retweet compared to the number of users who tweet.

**Figure 3.**
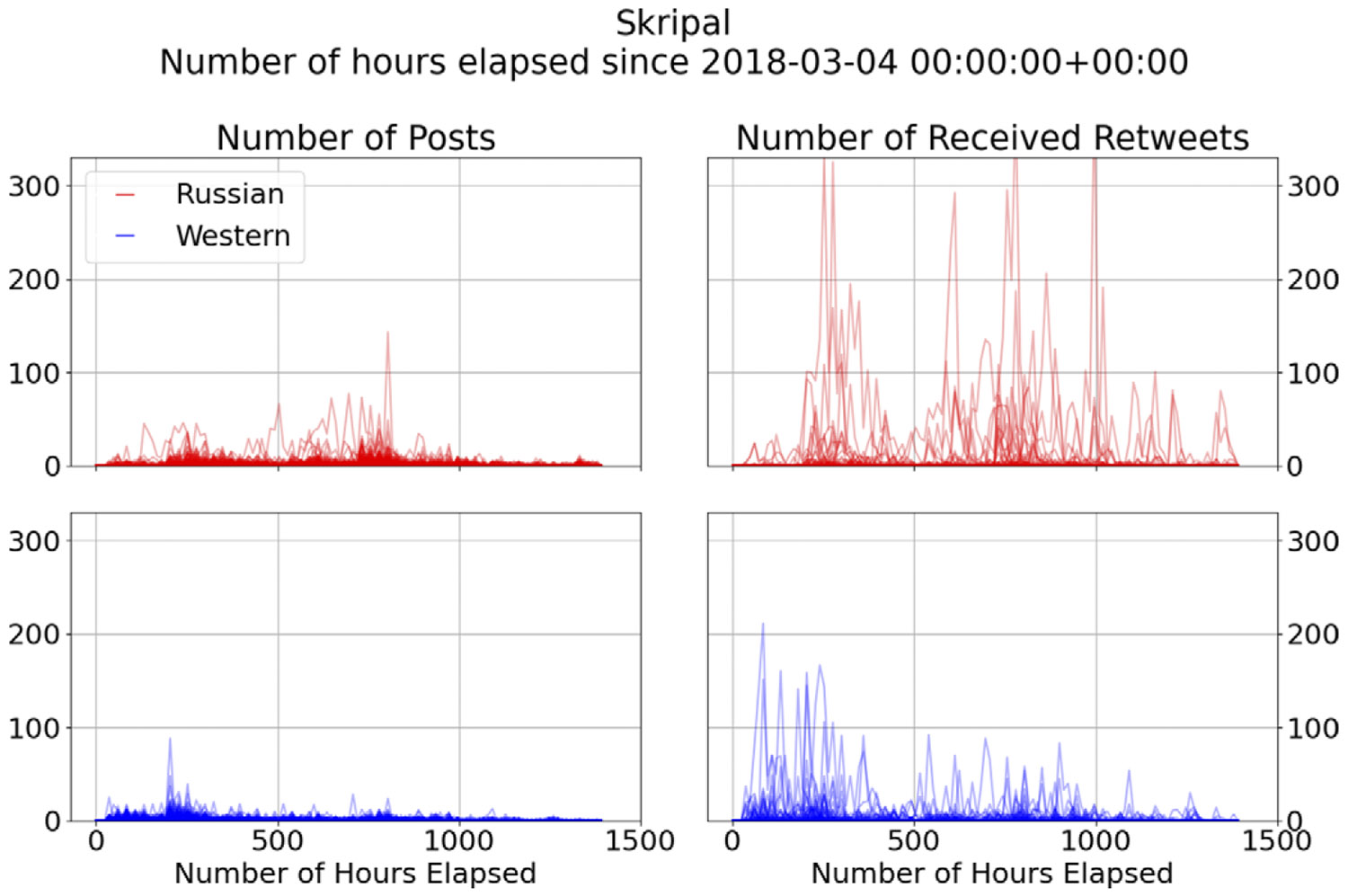
Skripal users’ total number of posts and retweets over time.

The raw total counts of tweets and retweets for each individual are observed discretely and are assumed to be generated by smooth curves, with observations taken every 12 hours within a compact interval. Figure 3 illustrates the different peaks observed in distinct communities, namely pro-Russian and pro-Western, where the number of engagements rapidly increases at varying times following the event.

Overall, the pro-Russian group exhibits a higher volume of posting in Skripal incidents, and they engage in even more retweeting compared to the pro-Western group. To be specific, the pro-Russian communities employ a strategy of amplifying the campaign messages through periodic, extremely high volumes of retweets. For instance, this volume can be as much as six times greater than that of the pro-Western group, particularly noticeable 10 days after the event occurrence. This same pattern repeats itself approximately 42 days after the event took place. The pro-Russian group displays multiple peaks in their engagement, and the detection of these peaks can be derived from the derivative of the engagement function at specific times.

#### 2.2.2. Functional data analysis

We assume that the functional curves represented by the number of posts and retweets denoted as $X_{i}(t)$ over time have a dense design. These curves are defined on the closed interval [0, 60], where i=1,⋯,n. We consider these individual curves as realizations of a stochastic process X(t) with a mean function μ(t) and covariance Σ. The total number of subjects is n=547 for the posts curves in both cases, n=272 for Skripal retweets, and n=84 for Bucha Crimes retweets.

The mean function for each group can be estimated using a sample mean function, and the covariance function can be estimated from the sample covariance as follows (J. O. Ramsay and Silverman, 2005):

(2.1) $$\bar{X}(t)=\frac{1}{n}\sum_{i=1}^{n}X_{i}(t)$$

(2.2) $$Cov_{X}(s,t)=\frac{\sum_{i=1}^{n}\{X_{i}(s)-\bar{X}(s)\}\{X_{i}(t)-\bar{X}(t)\}}{n-1}$$

The objective of FPCA is to identify the projections that capture the maximum variance from the curves X(t), which represent deviations in the number of posts compared to the mean curve. Here, t∈𝒯. We can define the covariance function as follows:

$$\Sigma (s,t)=E[(X_{i}(s)-\mu (s))(X_{i}(t)-\mu (t))]$$

and

(2.3) $$\Sigma f(t)=\int_{𝒯}\Sigma (s,t)f(s)ds$$

where $f(t)\in L^{2}[𝒯]$. Functional principal component (FPC) $\phi_{k}$ are the eigenvectors of the covariance operator Σ such that

(2.4) $$\Sigma \phi_{k}=\lambda_{k}\phi_{k}$$

where $\lambda_{1}>\lambda_{2}\cdots >\lambda_{k}$ such that independent random variables $\xi_{ik}∼Normal(0,\lambda_{k})$. Functions $\phi_{k}$ are the unitary, orthonormal basis and Σ needs to be continuous, symmetric and non-negative definite. Karhunen–Loève expansion of the random function $X_{i}(t)$ with a finite truncation can be represented as

(2.5) $$X_{i}(t)=\mu (t)+\sum_{k=1}^{K}\xi_{ik}\phi_{ik}(t),t\in 𝒯$$

where $\phi_{k}$ is the FPCs and zero mean random variables $\xi_{k}$ is the FPC scores (Rice and Silverman, 1991; Silverman, 1996). The FPCA literature is rich for densely observed continuous functional data such as Bali et al. (2011) and Ramsay and Silverman (2005) and see Shang (2014) for a complete list. We used regression-based method to estimate smooth mean function and fast covariance estimation based on penalized splines proposed by Xiao et al. (2016).

**Figure 4.**
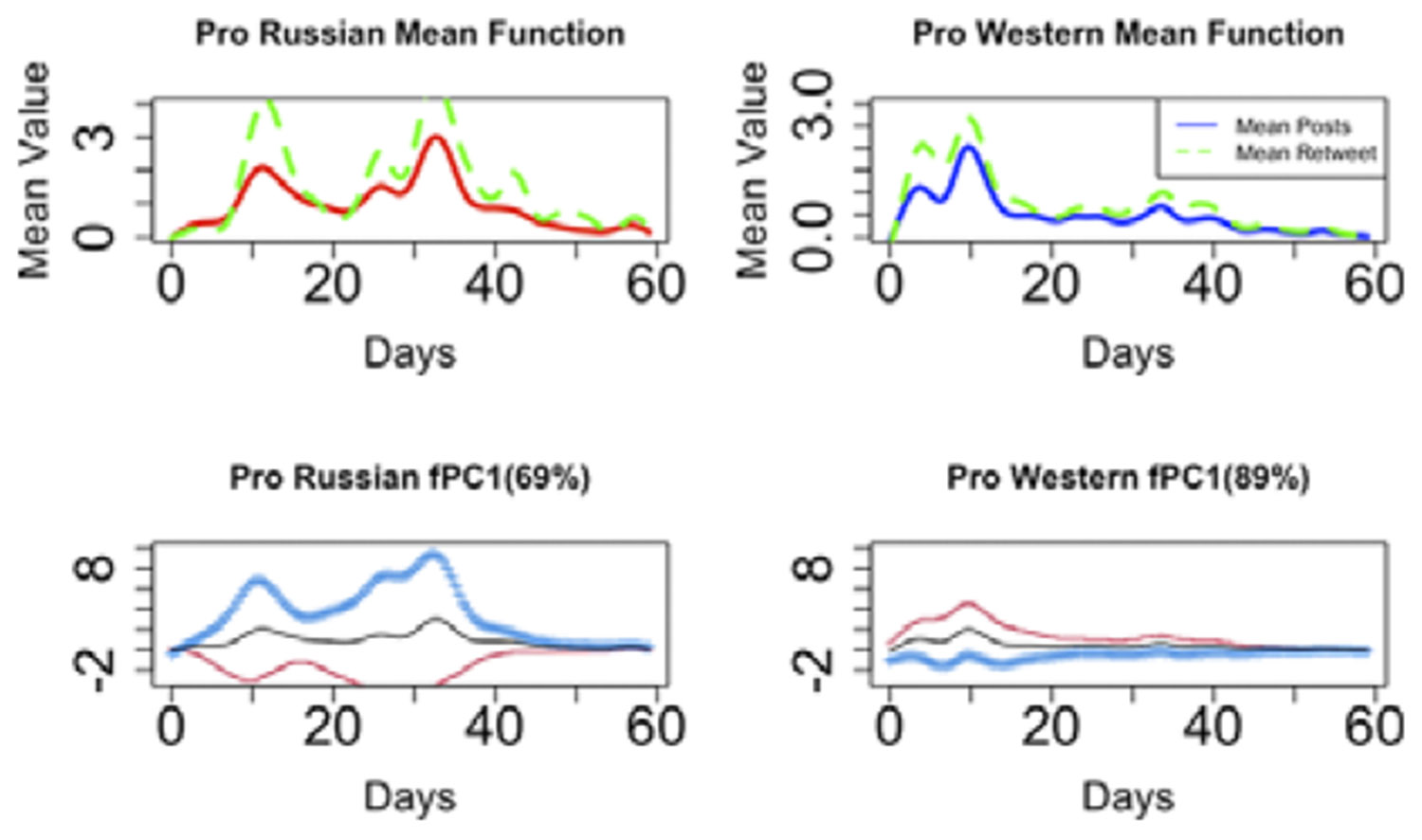
Top: Mean posts function and mean retweets over time for skripal event, bottom: First fpc and one standard deviation over time by communities.

As shown at the top of Figure 4, pro-Russian members exhibit a tendency to engage in high-volume tweeting and retweeting around the 12-day mark following the Skripal event, just before the occurrence of the second peak approximately 35 days in. In contrast, the pro-Western group demonstrates more active posting, showing an almost 60% increase compared to the first peak observed in pro-Russian members. The mean curve reaches its initial peak slightly later than the pro-Russian group, occurring at around 10 days.

In general, the pro-Russian groups exhibit more variability compared to the pro-Western groups in the Skripal case. The first FPC for pro-Russian data only captures 69% of the variability, while the first FPC for pro-Western data can account for 89% of the variance, as illustrated in the lower part of Figure 4. Users in the pro-Western group engage in almost identical ways with similar variabilities over time. In contrast, users in the pro-Russian group strategically exchange information based on the impact of the incident and the narratives surrounding it. This observation aligns with our earlier descriptions. We assume that the mean curve reflects the overall behavior of each community, with individual deviations being captured by the FPC scores. Consequently, we can cluster users based on these scores, and the FPCs elucidate the primary sources of variation.

The curves representing posts and retweets both exhibit their first peak at a similar time following the occurrence of the event, regardless of the group or the case. A significant insight derived from this discovery is that information pertaining to topics like this appears to undergo exponential amplification on social media platforms when there is a concurrent increase in both high-frequency posts and high-frequency retweets. This observation underscores the importance for response teams to closely monitor specific topics under these conditions and take action promptly if any disinformation is detected.

#### 2.2.3. Adaptive density peak detection

Given the varying peaks observed at different times among different groups, we employed multivariate functional data clustering with adaptive density peak detection, as detailed by Ren et al. (2023). In this approach, the first feature of each group comprises the post curves, while the second feature consists of the retweets curves. This method allows us to quantify the distinctions between the two groups by considering these two functional curves simultaneously, rather than examining them sequentially without accounting for the interdependence between the post curve and the retweets curve. Both of these aspects influence the ultimate outcome of information flow.

Functional data clustering using adaptive density peak detection is a method that employs a functional k-nearest neighbor density estimator based on L2 distance between raw functional curves. You can find more detailed information in the reference by Ren et al. (2023). Notably, this approach is computationally efficient, as it does not necessitate an iterative process, as outlined by Ren et al. (2023).

The clustering process relies on two key metrics: the local density $\hat{f}(x_{i};H)$ and a “minimum” distance ${\hat{\delta }}_{i}$ of the multivariate functional curve. Cluster centers are defined as points where both their local density and “minimum” distance are exceptionally large.

(2.6) $$\hat{f}(x_{i};H)=n^{-1}{|H|}^{-1∕2}\sum_{i=1}^{n}K\left(H^{-1∕2}(x_{i}-x_{j})\right)$$

and

(2.7) $${\hat{\delta }}_{i}=\underset{j:\hat{f}(x_{i};H)<\hat{f}(x_{j};H)}{\text{min}}d(x_{i},x_{j})$$

In our example, $x_{i}={\left(posts_{i1},retweets_{i2}\right)}^{T}\in R^{2}$, $H_{2\times 2}$ is a symmetric positive definite matrix named the bandwidth matrix, and K(⋅) is a two-variate kernel function satisfying ∫K(x)dx=1, the minimum distance measure $\delta_{i}$ is the minimum distance between the point i and any other point j with higher density $\hat{f}(x_{i};H)$ (Ren et al., 2023).

After identifying the cluster centers for each group, the remaining curves are allocated to these cluster centers using the criterion of minimum nearest distance, as described in the work by Ren et al. (2023).

**Figure 5.**
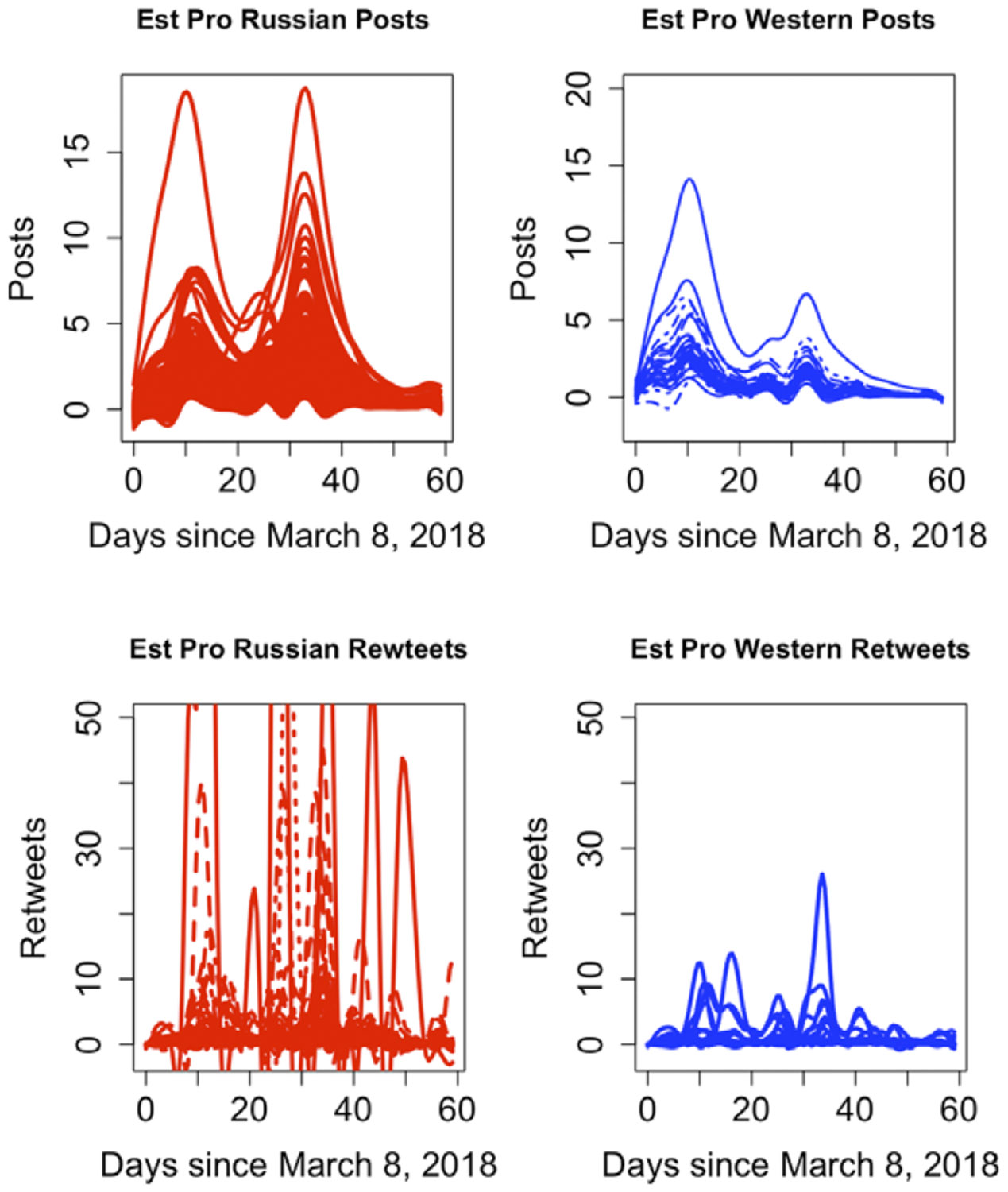
Skripal data, top: Estimated posts function by communities over time, bottom: Estimated retweets function over time by communities.

After clustering using the adaptive density peak method, we can recover the posts curves and retweets curves for Skripal within their respective communities. In terms of the estimated posts curves, the pro-Russian group reaches a higher peak later, while the pro-Western group achieves a higher peak first after the event for Skripal incident. As illustrated in Figure 5, the estimated pro-Russian posts curves exhibit two peaks, with a lower peak around 12 days and a higher peak around 35 days. Conversely, pro-Western members display two peaks, with a relatively higher one at around 10 days and a lower peak around 35 days. The recovered retweet curves echo the variability within the pro-Russian group we found earlier in Figure 4.

The Adjusted Rand Index (Rand, 1971), which measures the similarity between two clustering, is 0:78 for Skripal incident, although it is important to note that this result is contingent on the data available.

In summary, the estimated posts curves exhibit less variability in phase shift when compared to the retweets curves. The estimated retweets curves reveal multiple peaks in both the pro-Russian and pro-Western groups. In particular, the peaks in the pro-Russian group consistently surpass those in the pro-Western group throughout the 60-day observation period. The varying phase shifts observed across all subjects confirm that user engagement with a specific tweet is mirrored by the flow of information and activities within or outside the community.

For this specific dataset, engaging in discussions on or before the 12-day mark, when the pro-Russian groups have either just reached or are approaching their first peak, may potentially reduce the influx of information related to the campaign into the pathway for Skripal.

#### 2.2.4. Canonical correlations

FCCA is a tool used to quantify correlations between pairs of observed random curves when a sample is available, as detailed in references by Krzyśko and Waszak (2013) and Ramsay and Silverman (2005). FCCA aims to identify associations between two functional curves, and the strength of the relationship between pairs of variates is measured using canonical correlation coefficients ($R_{c}$) as described by Dattalo (2014).

To calculate the functional canonical correlation, we seek to maximize the penalized squared sample correlation function ${\mathrm{ccorsq}}_{\lambda }(\xi ,\eta )$ in terms of unknown functions ξ and η. This function can be expressed as follows:

(2.8) $$\frac{cov{\left(\left(\int \xi X_{i},\int \eta Y_{i}\right)\right)}^{2}}{\left\{var\left(\int \xi X_{i}\right)+\lambda {\left\|D^{2}\xi \right\|}^{2}\right\}\left\{var\left(\int \eta Y_{i}\right)+\lambda {\left\|D^{2}\eta \right\|}^{2}\right\}}$$

where $X_{i}$ is the posts function for each individual, $Y_{i}$ is the retweets function for each individual, the roughness of a function f is its integrated squared curvature ${\left\|D^{2}f\right\|}^{2}=\int {\left(D^{2}f\right)}^{2}$ (Ramsay and Silverman, 2005). The regularization is used to improve the conditioning of the variance matrices (Ramsay and Silverman, 2005).

In our study, our aim is to measure the dependence of the total number of tweets on retweets within different communities. As observed in the work by Geissler et al. (2023), the propagation of pro-Russian support on social media, along with the dissemination of pro-Russian messages and its amplification, varies over time, with the majority of these activities occurring at the beginning of the invasion.

Differences in the co-varying effects between the two groups can be assessed through the examination of the first three canonical correlations. Specifically, for the pro-Russian group, the first three canonical correlations are 0:117,0:028, and 0:011, whereas for the pro-Western group, the first three canonical correlations are 0:380,0:129, and 0:048. To be specific, the first canonical correlation from the pro-Western group is nearly four times stronger than that of the pro-Russian group for the Skripal incident.

## 3. Group specific intervention versus population wise response

**Figure 6.**
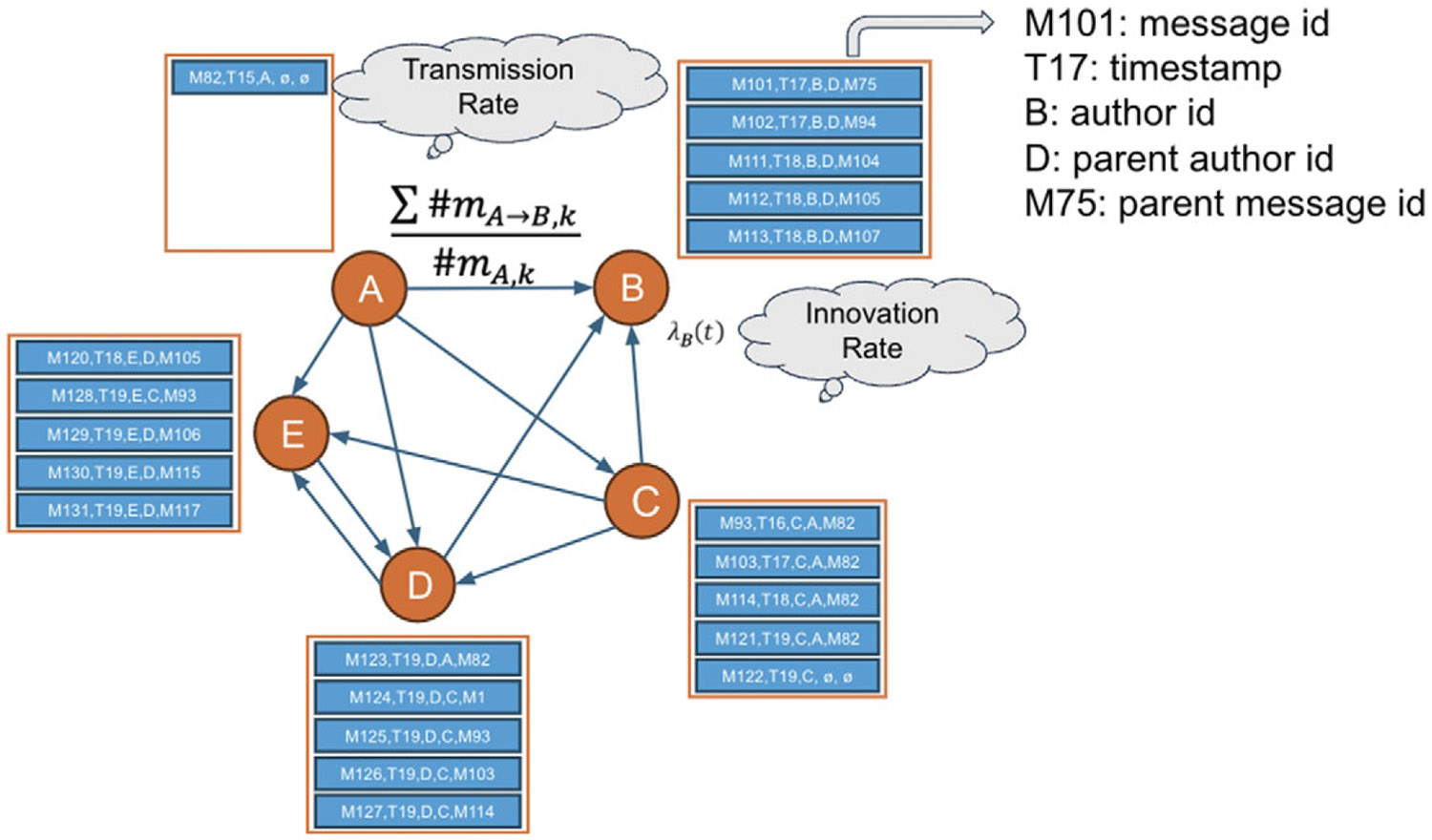
Simulation of information diffusion.

The previous analysis provides insights into differentiating communities. To illustrate the effectiveness of intervention strategies, we utilize simulated data generated through a model based on real data from the Skripal case. The threshold rate for incoming information is adopted from Rodriguez et al., 2014. Parameters such as the rate of tweets and retweets in the simulation are estimated from the real data, and the group characteristics of the pro-Russian and pro-Western communities are controlled by the properties identified in the previous section. To be specific, Figure 6 is the main idea of simulating information spread and influences.

The innovation rate (rate of tweets) of B is defined as

$$\lambda_{B}(t)=R_{B}(t\;\text{mod}\;24)+R_{Exo}(t)$$

where $R_{B}$ is the average tweets for each hour of the day of the 24 hours and $R_{exo}(t)$ indicates the exogenous event impact over the observational time.

The imitation rate (rate of retweets/replies), indicates how many message from A will B reply to,

$$\lambda_{A\to B}(t)=\frac{\sum_{k=1}^{T}\#m_{A\to B,k}}{\#m_{A,T}}\#m_{A}(t)$$

where $\#m_{A\to B,k}$ is the number of the messages by B that are replies to A at the timestep k, $\#m_{A,T}$ is the number of messages of A at T, and $\#m_{A}(t)$ is the hourly count of the number of messages of A.

Each author at timestep t will observe the messages from neighbors and decide to retweet. If the observed number of messages are over a certain limit, the user’s capacity to consider and reply to all the received messages become limited, due to biological and social limitations (Gunaratne et al., 2020). A user is considered as overloaded if $O_{t}=R_{t}-30>0$ where $R_{t}$ is the total number of observed messages. $O_{t}$ represents the overload at the time t which occur when $R_{t}$ exceeds 30 (Rodriguez et al., 2014; Gunaratne et al., 2020). The effective number of messages replied to, when there is an overload, is reduced to an amount given by $O_{t}^{\alpha }$ where α∈(0,1) is a parameter that indicates the reduction effect caused by overload. An author replies to each neighbor by considering the recent history of their own interactions. During an overload, a subset of recently interacted users is prioritized for replies. Otherwise, retweets for neighbors are prioritized based on the transmission rate for each neighbor and the number of messages received by the neighbor. Both the number of retweets and tweets are randomly generated from a Poisson distribution with known transmission and innovation rates.

### 3.1. Simulation

We simulated two communities based on the characteristics from two communities displayed in Figures 3 and 4.

**Figure 7.**
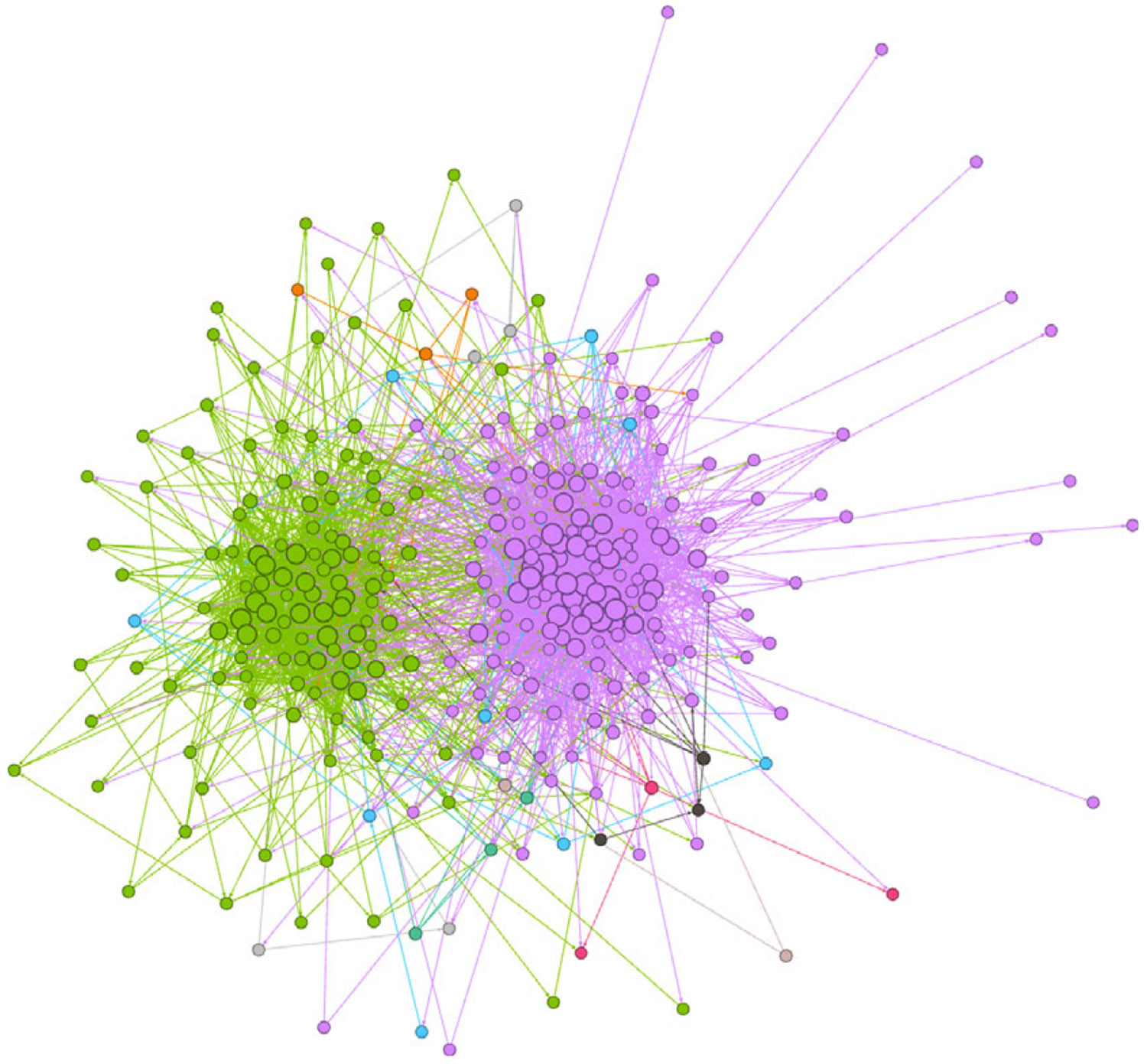
Simulation of two social network communities.

Our goal is to compare the effects of removing influential nodes from the network. We highlight the importance of the criteria for banning. Our premise is that selecting from an identified group of user accounts is effective than identifying a general set of influential nodes for removal. Therefore, we design our experimental setup such that we can run an information diffusion simulation through our proposed model, then identify influential nodes based on different criteria, and finally rerun the simulation on the same network with those nodes removed. We then compare the results based on each criterion. We use the directed scale-free network generation algorithm by Bollobás et al. (2003) with β=0.95 and $\delta_{in}=\delta_{out}=0.1$ to generate two communities with 2 : 1 ratio of nodes between the pro-Western and pro-Russian groups, which approximates the ratio found in the Skripal dataset. The communities are interconnected with a probability of 0:01. The network size used for the experiments was 600 nodes (200 pro-Russian and 400 pro-Western). A smaller example network of 300 nodes is shown in Figure 7.

**Figure 8.**
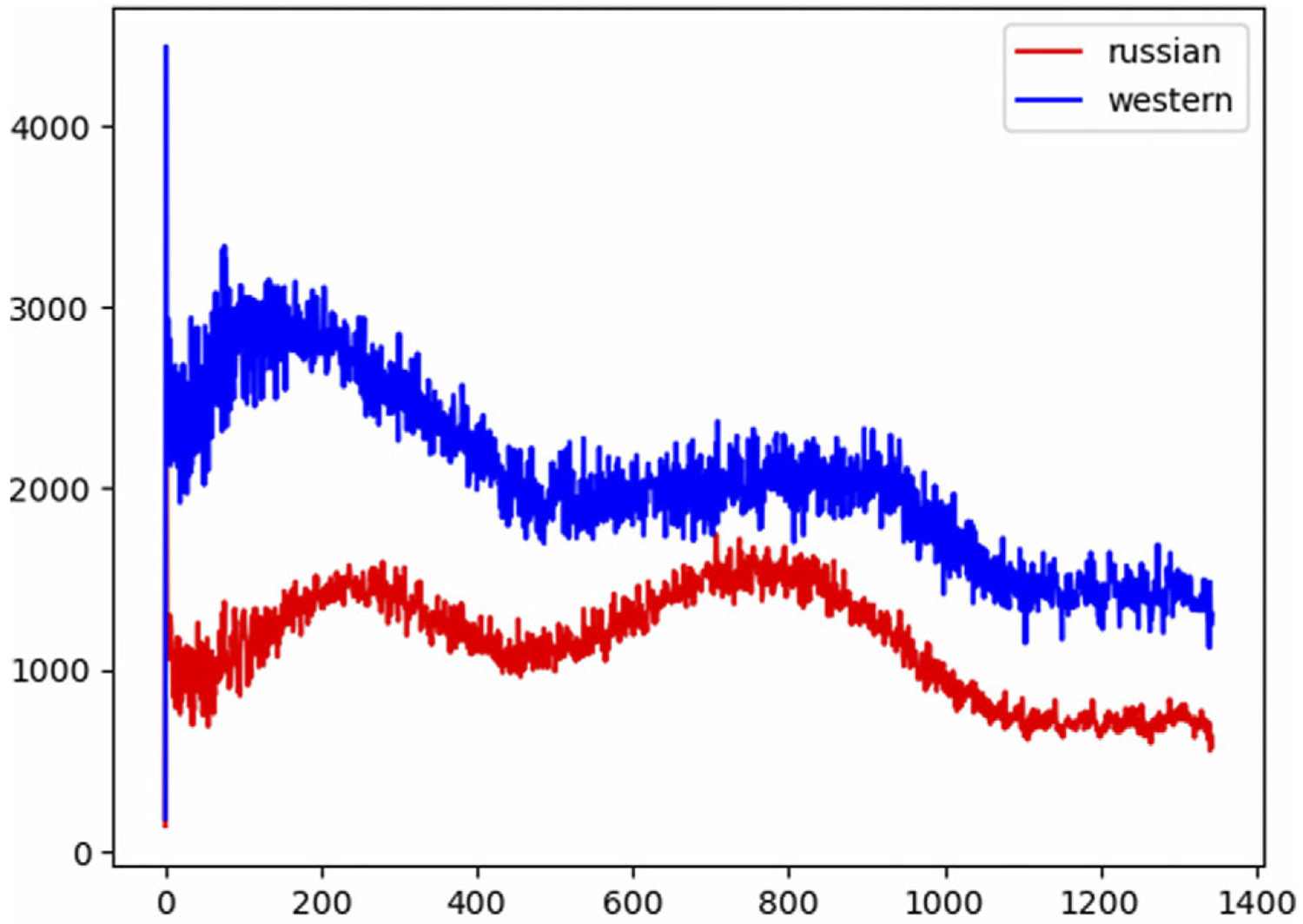
Campaigns without interventions.

Figure 7 is generated using the Yifan Hu layout from Gephi, including 300 nodes, where 100 belong to one community and 200 to another, with a connection probability of 0:01 between the two communities to simulate our Russian and Western communities. The nodes are sized based on out-degree ranking, with a minimum size of 5 and a maximum size of 10. The network’s diameter is 8, its modularity is 0:372 based on community detection, and the average path length is 3:228.Figure 8 shows tweet volume over time without any intervention applied.

**Figure 9.**
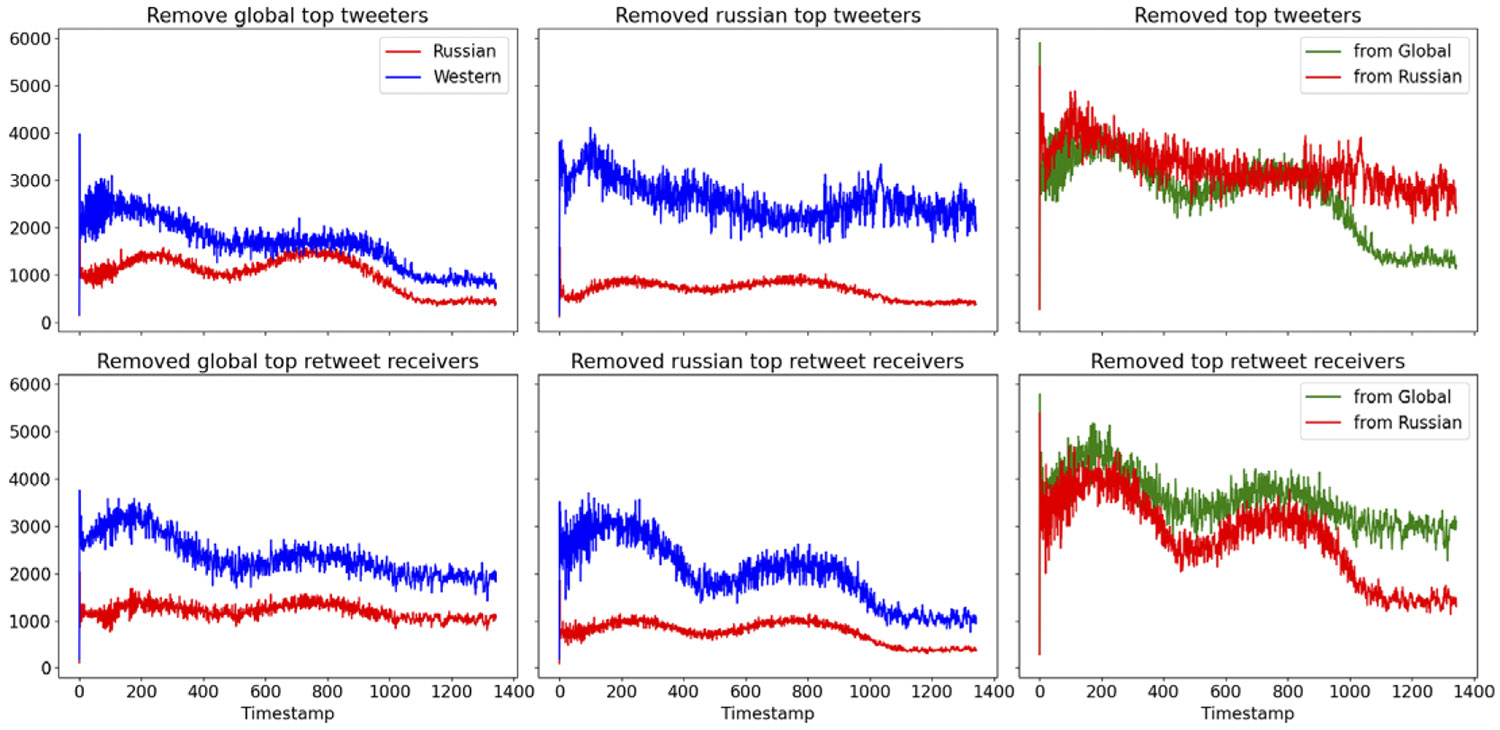
Effects of intervention strategies.

Figure 9 indicates the differences between node removal intervention strategies: global and community-based. We hypothesize that removing influential nodes from a targeted group is more effective in minimizing the spread of a growing campaign, specifically in terms of reducing the number of retweets in our context. While influence could be measured in many forms, in our experiment it was measured through the number of generated tweets and the number of received retweets. The top row of Figure 9 is related to the measure of top tweeters (users who generate the most tweets) and the bottom row is related to the users who were retweeted the most by others. The leftmost column shows the effect of removing the top 10% influential users from the entire population. The middle column demonstrates the effect of removing the top 10% of influential users from the target group (pro-Russians). In both columns, blue represents the simulated Western group, and red indicates the simulated Russian group. Both types of removal reduce the overall number of tweets and retweets, reflecting the impact of efforts to mitigate the growth of the topic in terms of tweets and its amplification in terms of retweets.

The rightmost column compares the effect of each intervention measure on the total number of messages, tweets, and retweets in the system. As shown in the top-right figure, global intervention can maximize the reduction of message innovation, while community-based intervention, as shown at the bottom of the figure, dramatically reduces message propagation in our context.

#### 3.1.1. Consistency across scenarios

**Figure 10.**
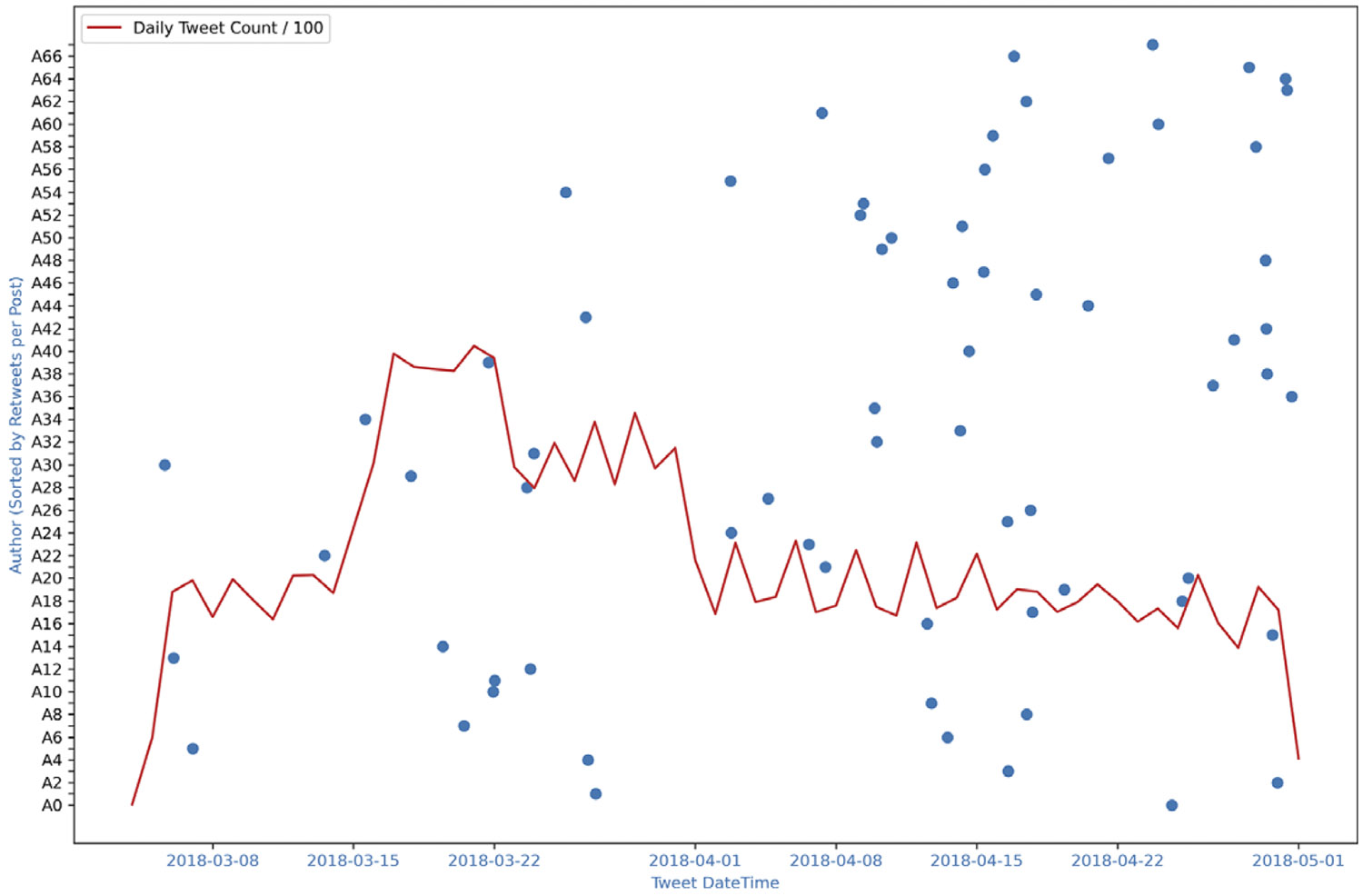
Skripal case: The last tweet date-time of top 68 users and tweet volume. Blue dots represent the timestamp of the last tweet of author. Red line is the total Tweet volume. Authors sorted such that A0 has the highest retweets per post (most influential) and A67 has the lowest.

We hypothesize that the departure of influential users leads to a significant decline in overall conversation volume. To test this, we first examined the Skripal dataset. To test this, we first examined the Skripal dataset. Figure 10 plots the last tweets of top users relative to the total tweet volume over time. Users are ordered by their average retweets per post, a proxy for influence. As highly influential users stop posting (particularly those in the lower-left region of the graph) the overall tweet volume declines. A paired comparison of daily tweet counts (i.e., average tweet volume per day) before and after each top author’s final post confirmed this pattern. The Wilcoxon signed-rank test indicated a significant decrease in activity (Wilcoxon statistic: 2088.0 *p*-value: 1.12e-08).

**Figure 11.**
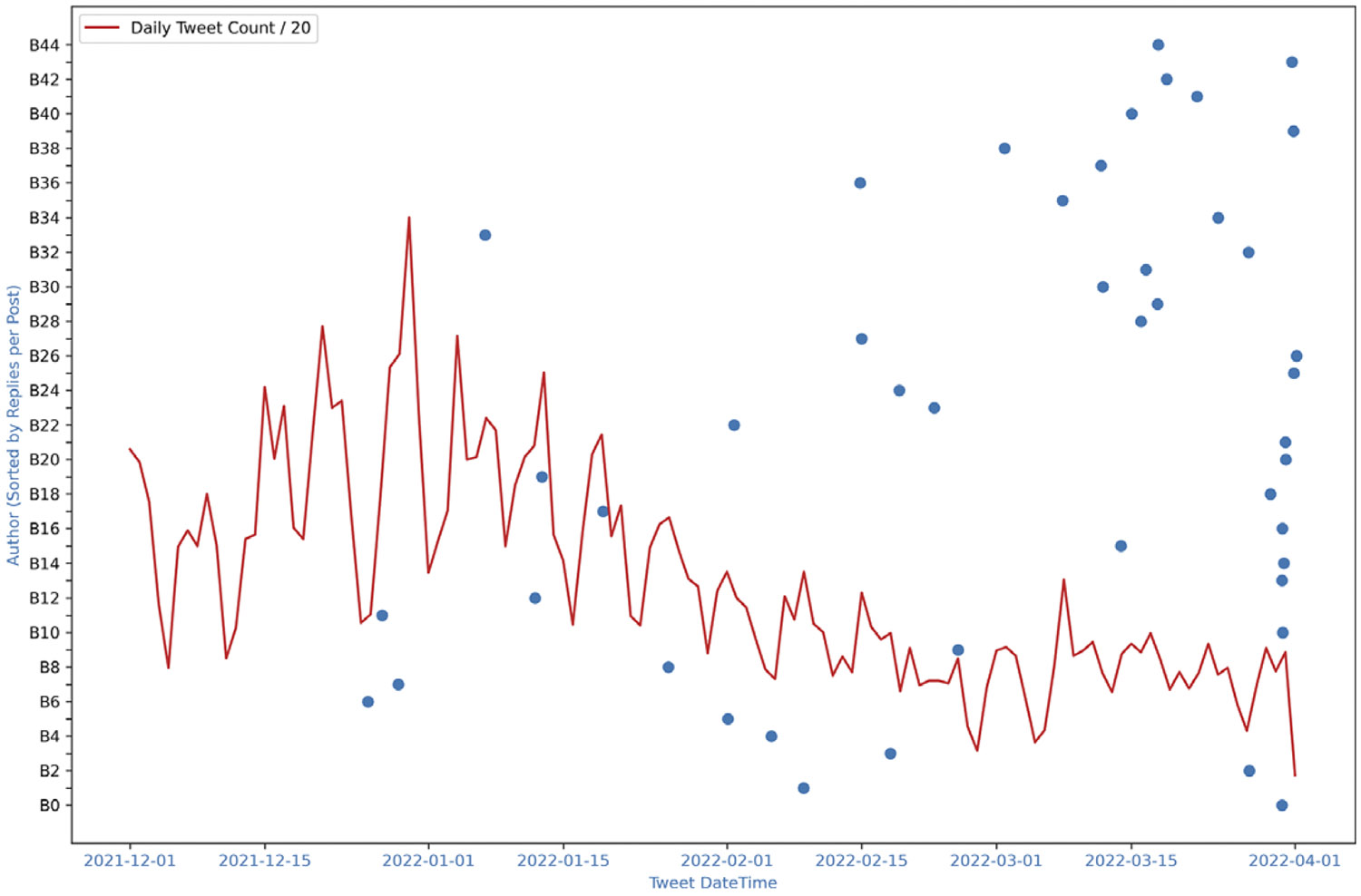
COVID19 case: The last tweet date-time of top 45 users and tweet volume. Blue dots represent the timestamp of the last tweet of a given author. Red line is the total Tweet volume. Authors sorted such that B0 has the highest replies per post (most influential) and B44 has the lowest.

To assess the consistency of this effect, we extended our analysis to a Twitter dataset containing the hashtag #COVID19, collected between January 1 and April 1, 2021, from a set of 3900 users. While the dataset included multiple hashtags, we selected #COVID19 due to its high volume of activity. Figure 11 shows the last tweet times of top users with the overall tweet volume. A similar pattern emerges: highly influential users tend to leave around the peak of activity, coinciding with the subsequent decline in tweet volume. We applied the same statistical test to this dataset and obtained consistent results. The Wilcoxon signed-rank test indicated a significant reduction in tweet activity following the departure of top nodes (Wilcoxon statistic: 990.0 *p*-value: 5.68e-14), confirming that average tweet volume before their exit is significantly greater than afterward.

Together, these results demonstrate a consistent relationship across different contexts. When top users exit a conversation, overall participation declines significantly. This provides empirical support for our broader argument that targeting interventions at community leaders or highly influential nodes can have a strong effect on the volume of information flow.

## 4. Discussion

This study focuses on important distinctions in the dynamics of information diffusion on social media, particularly when comparing community-based interventions with population-wide approaches. Our analysis shows that community-based interventions have a more significant and targeted impact on information dissemination within specific groups in terms of amplification, while population-wide approaches tend to distribute information more uniformly but with less intensity, and work more effectively in mitigating overall innovation on a given topic.

From a decision-maker’s standpoint, the results of this article mean that if they are interested in disrupting information flow or protecting information flow from an adversary, then they need to focus more on community properties of the network as opposed to individual, node-level properties. Policymakers must decide whether their priority is to reduce the strength of diffusion in key groups or to dampen the overall spread across society. In addition, counternarratives may require a hybrid approach that contains a targeted effort aimed at high-risk groups and a population-wide signal for the general population. This also implicates how community-level moderation tools are especially valuable (e.g., moderation within subreddits) to dampen amplification of harmful narratives. Further, platforms may need to establish broad systematic rules to ensure consistent mitigation of information risks across the population (e.g., the way StackOverflow enforces question standards to maintain the quality of its information space).

The use of FDA allowed us to capture the temporal behavior of social media users, particularly during high-profile incidents like the Skripal/Novichok case. By examining time-dependent metrics such as posts and retweets, we gained insights into how communities react in real time to such events. These temporal dynamics demonstrated that social media communities are highly responsive to external triggers, often leading to bursts of activity that can accelerate the spread of particular narratives.

We quantified disparities between communities using three distinct methods, uncovering patterns that suggest each community adopts different strategies to promote narratives. Some communities acted as echo chambers, amplifying a single message, while others fostered a more diverse range of content, allowing for broader discussions. This variation in narrative promotion indicates that interventions must be tailored to community characteristics to be effective.

When comparing community-based interventions with population-wide responses, the former showed a more significant effect in guiding the direction and scope of information diffusion. Targeted interventions, which directly engage with key community influencers, led to more sustained engagement and a higher degree of message penetration. In contrast, population-wide interventions were more diffuse and resulted in lower engagement per individual community, suggesting that a broad approach can dilute the impact of a message.

The study highlights the importance of recognizing the unique structure of social media communities when designing intervention strategies. Community-based interventions have the advantage of lever-aging existing networks and amplifying messages through trusted channels, making them more efficient in fostering information spread. Meanwhile, population-wide approaches can still be valuable for ensuring widespread exposure but may be less effective in influencing deep engagement or shifting public perception within specific groups.

Ultimately, our findings emphasize the need for a balanced strategy, in which both community-specific and broad interventions are employed depending on the objectives. For policymakers and organizations looking to influence public discourse on social media, understanding these differences is crucial for maximizing the effectiveness of their communication strategies.

Finally, it is important to note that our intervention estimates are counterfactual simulations. Future work will exploit natural experiments (e.g., staggered policy shifts or platform enforcement episodes) using event-study, synthetic-control, and difference-in-differences designs. These experiments will be used to test whether observed changes in community-level exposure and cascade metrics align with simulated predictions.

## Figures and Tables

**Table 1.** Users summary: Skripal

Category	Posts	Retweets	% of Retweet
Pro-Russian	183	113	0.38
Pro-Western	364	159	0.30

## Data Availability

The data that support the findings of this study are openly available at https://doi.org/10.5281/zenodo.17231606.

## Policy Significance Statement

This research highlights the importance of tailored interventions for effective information control on social media. By comparing community-specific strategies with population-wide responses, the study shows that targeted approaches within specific social media groups can be more effective in managing the spread of disinformation and manipulated messages in our context. Policymakers can benefit from these insights when developing strategies to combat misinformation, as interventions tailored to distinct communities may result in more precise outcomes due to the nature of the manipulated campaign. Understanding how information flows differently across groups allows for better decision-making when shaping public opinion and discourse. This research provides valuable guidance for creating policies that address the complexities of information diffusion.
